# Identification of a novel immune-related transcriptional regulatory network in sarcopenia

**DOI:** 10.1186/s12877-023-04152-1

**Published:** 2023-07-31

**Authors:** Xianzhong Zhang, Guanglou Zhu, Fengmin Zhang, Dingye Yu, Xuyang Jia, Bingwei Ma, Weizhe Chen, Xinyu Cai, Lingzhou Mao, Chengle Zhuang, Zhen Yu

**Affiliations:** 1 Department of Gastrointestinal Surgery, Shanghai Tenth People’s Hospital, Tongji University School of Medicine, Middle 301 Yanchang Road, Shanghai, 200072 China; 2 Department of General Surgery, Shanghai Minimally Invasive Surgery Center, Ruijin Hospital, Shanghai Jiao Tong University School of Medicine, Shanghai, China; 3 Department of Endocrinology and Metabolism, Shanghai Tenth People’s Hospital, Tongji University School of Medicine, Shanghai, China; 4 Department of Orthopedics, Shanghai Tenth People’s Hospital, Tongji University School of Medicine, Shanghai, China; 5 Colorectal Cancer Center, Shanghai Tenth People’s Hospital, Tongji University School of Medicine, Shanghai, China

**Keywords:** Sarcopenia, RNA-seq, Transcription factors, Immune gene sets, Regulatory network

## Abstract

**Background:**

Sarcopenia is highly prevalent in elderly individuals and has a significant adverse effect on their physical health and quality of life, but the mechanisms remain unclear. Studies have indicated that transcription factors (TFs) and the immune microenvironment play a vital role in skeletal muscle atrophy.

**Methods:**

RNA-seq data of 40 muscle samples were downloaded from the GEO database. Then, differentially expressed genes (DEGs), TFs(DETFs), pathways(DEPs), and the expression of immune gene sets were identified with limma, edgeR, GO, KEGG, ORA, GSVA, and ssGSEA. Furthermore, the results above were integrated into coexpression analysis by Pearson correlation analysis (PCA). Significant coexpression patterns were used to construct the immune-related transcriptional regulatory network by Cytoscape and potential medicine targeting the network was screened by Connectivity Map. Finally, the regulatory mechanisms and RNA expression of DEGs and DETFs were identified by multiple online databases and RT‒qPCR.

**Results:**

We screened 808 DEGs (log2 fold change (FC) > 1 or <  − 1, *p* < 0.05), 4 DETFs (log2FC > 0.7 or <  − 0.7, *p* < 0.05), 304 DEPs (enrichment scores (ES) > 1 or <  − 1, *p* < 0.05), and 1208 differentially expressed immune genes sets (DEIGSs) (*p* < 0.01). Based on the results of PCA (correlation coefficient (CC) > 0.4 or <  − 0.4, *p* < 0.01), we then structured an immune-related network with 4 DETFs, 9 final DEGs, 11 final DEPs, and 6 final DEIGSs. Combining the results of online databases and in vitro experiments, we found that PAX5-SERPINA5-PI3K/Akt (CC ≤ 0.444, *p* ≤ 0.004) was a potential transcriptional regulation axis, and B cells (*R* = 0.437, *p* = 0.005) may play a vital role in this signal transduction. Finally, the compound of trichostatin A (enrichment = -0.365, specificity = 0.4257, *p* < 0.0001) might be a potential medicine for sarcopenia based on the PubChem database and the result of the literature review.

**Conclusions:**

We first identified immune-related transcriptional regulatory network with high-throughput RNA-seq data in sarcopenia. We hypothesized that PAX5-SERPIAN5-PI3K/Akt axis is a potential mechanism in sarcopenia and that B cells may play a vital role in this signal transduction. In addition, trichostatin A might be a potential medicine for sarcopenia.

**Supplementary Information:**

The online version contains supplementary material available at 10.1186/s12877-023-04152-1.

## Background

Sarcopenia contributes to the accelerated loss of muscle mass and function with ageing and associated with increased risk of falls, postoperative complications, frailty, and mortality [1, 2]. To date, the mechanisms of sarcopenia remain largely unknown, and no effective drugs have been approved for clinical treatment [3]. Lifestyle interventions, especially exercise and nutritional supplementation, prevail as the mainstays of treatment [4]. More efforts need to be made to explore the pathogenesis of sarcopenia, as well as to develop novel treatments.

Transcription factors (TFs) can specifically interact with the cis-acting elements of eukaryotic genes and control the rate of genetic information from DNA to mRNA [5]. A Number of studies have indicated that TFs are actively involved in skeletal muscle diseases [6, 7]. As the immune microenvironment also plays a vital role in the occurrence and development of sarcopenia [8]. It has been proven that aberrant interactions among skeletal muscle fibres and immune cells contribute to the atrophy of skeletal muscle and decreased muscle function [9]. Abnormal biological behaviours in the muscle fibre microenvironment, such as inflammation and hypoxia, have also been attributed to the aberrant expression of TFs [10, 11].

Here, we constructed an inclusive immune related transcriptional regulatory network in sarcopenia, which included the differentially expressed TFs, downstream genes, pathways, and immune-related events and cells. In this study, we first obtained the RNA-seq profiles from the Gene Expression Omnibus (GEO) database and obtained the differentially expressed genes (DEGs) by the limma package. Then, the differentially expressed TFs (DETFs) were identified by the edgeR package. We next explored the differentially expressed pathways (DEPs) with overrepresentation analysis (ORA) and Gene Set Variation Analysis (GSVA). Single-sample gene set enrichment analysis (ssGSEA) was also performed to identify the differentially expressed immune gene sets (DEIGSs) in sarcopenia. After that, we proposed a systematic and innovative regulatory network with an immune signature in sarcopenia by using Pearson correlation analysis(PCA) and Cytoscape. Finally, both online verification (NCBI, UCSC and JASPAR) and in vitro experiments were conducted to confirm our findings. Moreover, Connectivity Map (C-Map) was exploited to screen possible compounds targeting this network and develop potential drugs for sarcopenia.

## Methods

### Data acquisition

RNA-seq data of 40 primary vastus lateralis muscle samples were downloaded from the GEO database (GSE113660, https://www.ncbi.nlm.nih.gov/geo/), which included 20 cases with sarcopenia and 20 age-matched and sex-matched healthy controls. Sarcopenia was defined based on harmonized consensus clinical definitions of the 2019 Asian Working Group for Sarcopenia Consensus (AWGS) [12].

### Identification of DEGs, DETFs and functional annotation

“Limma” (version 3.50.3, https://bioinf.wehi.edu.au/limma/) was used to identify DEGs. Genes with log2fold change (FC) > 1or > 1 or <  − 1 and *p* value < 0.05 were regarded as DEGs. Then, sequencing data for TFs were retrieved from the Cistrome database (http://cistr ome.org/ ), and DETFs were identified with *p* < 0.05 and log2FC > 0.7 or <  − 0.7. Functional annotation of DEGs was analysed using Gene Ontology (GO) and Kyoto Encyclopedia of Genes and Genomes (KEGG) datasets to examine potential mechanisms of sarcopenia, and *p* < 0.05 was considered statistically significant. The bar plot of GO and circle plot of KEGG pathway annotation were generated with “enrichplot” (version 1.10.2), “GOplot” (version 1.0.2) and “ggplot2” (version 3.3.5).

### Identification of DEPs

The ORA bubble image was generated to show the DEPs by the R package according to the DEGs results. The DEPs were also qualified by GSVA(version 1.42.0) based on the MSigDB v7.5.1 (https://www.gsea-msigdb.org/gsea/msigdb/genesets.jsp?collection=H). The DEPs identified by GSVA were defined as those with enrichment scores (ES) > 1 or <  − 1, and the negative and positive signs represented pathway downregulation and upregulation, respectively.

The identification of final DEGs, DEPs and DEIGSs by Pearson correlation analysis.

PCA was adopted to identify the final DEGs, final DEPs, and final DEIGSs, and correlation analysis between two variables was performed by the Pearson correlation coefficient (PCC). The formula for calculating the PCC is as follows:

$$r\left(X,Y\right) = \frac{\mathrm{Cov}\left(X,Y\right)}{\sqrt{\mathrm{Var}\left[X\right]\mathrm{Var}\left[Y\right]}}$$

The PCC is represented by the coefficient r, Cov(X,Y) represents the covariance between two variables, and Var[X] and Var[Y] represent the variance of two variables. The two variables were considered positively correlated (1 ≥ r > 0), uncorrelated (*r* = 0), or negatively correlated (-1 ≤ r < 0), and a *p* value < 0.05 represented a significant difference.

For the identification of final DEGs, the amount of gene expression for DETFs and DEGs was extracted from RNA-seq and input into the formula. The final DEGs were defined as those with an absolute value of PCC > 0.4 and *p* < 0.01. For the final DEPs, the amount of gene expression for final DEGs and ES of DEPs were extracted from RNA-seq and the results of GSVA analysis, respectively. Then, the PCC and *p* value of the DEPs and final DEGs were calculated. The final DEPs were defined as having an absolute value of PCC > 0.4 and *p* < 0.01. The resulting data were used to generate heatmaps and volcano plots using R software (version 3.5.3).

For the identification of final DEIGSs, first, ssGSEA was performed by the R package GSVA to assess the ES of immune cells and pathways for every single sample, and the quantity of 29 immune gene sets in the sarcopenia group (SP) and nonsarcopenia group (NSP) was evaluated by the CIBER-SORT algorithm. Then, PCA was used to calculate the correlation between immune gene sets and DEGs by evaluating the ES and the amount of gene expression. The DEIGSs were defined as having absolute values of PCC > 0.4 and *p* < 0.0106. The final DEIGSs were further identified by backtracking the corresponding DEIGSs of the final DEGs.

### Network construction and connectivity map analysis

Then, network was built by Cytoscape (version 3.7.1) based on the PCA results. Finally, the sarcopenia-related hypothesis built on the bioinformatics was displayed by a signalling diagram. The potential inhibitors of the network were screened by C-Map (build 02) (https://portals.broadinstitute.org/cmap/). The key medicines were identified with* p* < 0.01, and information of inhibitor compounds, such as chemical structural formula, biologic function, and clinical applications, was downloaded from the PubChem database (https://pubchem.ncbi.nlm.nih.gov/).

### Online database validation of the regulatory mechanism between the final DEGs and DETFs

For annotation of the final DEGs and DETFs, three algorithms (NCBI, UCSC and JASPAR) were used to repredict the transcriptional regulatory pattern among them. First, the promoter sequence regions of the final DEGs were obtained from the NCBI Gene database(https://www.ncbi.nlm.nih.gov/gene). Then, the target DETF binding site distribution in the target promoter regions was detected with the UCSC database(http://www.genome.ucsc.edu/). Finally, the JASPAR database(https://jaspar.genereg.net/) was used to predict the binding sites sequence and the relative score between the DETFs and final DEGs. The scan relative profile score threshold in the JASPAR database was set as 80%.

### Patients and groups

In total, 24 Chinese male participants aged 69–84 years with or without sarcopenia were recruited from a prospective cohort study approved by the Shanghai Tenth People’s Hospital Affiliated with Tongji University School of Medicine. In brief, patients who were diagnosed with low-energy hip fracture and planned to undergo hip replacement surgery within 3 days after hip fracture were eligible for participation in the study between October 2021 and December 2021. The diagnosis of sarcopenia was defined as low muscle strength (dominant hand grip < 28 kg) and low appendicular skeletal muscle mass (ALMi < 7.00 kg/m^2^) based on the 2019 AWGS [12].

### Body composition analysis and muscle strength test

On the day of admission, the body composition of participants was measured by using direct segmental multifrequency bioelectrical impedance analysis (DSM-BIA) equipment InbodyS10 (Inbody CO., Seoul, Korea) in a supine position. DSM-BIA was performed using a tetrapolar 8-point electrode system following standard protocols [13]. Lean body mass, fat mass and appendicular lean mass were calculated by the device based on the differences in electrical conductance.

The isokinetic strength of the participant’s dominant hand was measured using an electronic hand dynamometer (EH101; Camry, Guangdong Province, China) on the day of admission. The maximal value of three consecutive tests was recorded. Muscle strength was defined as the highest muscular force output (peak torque) in kilograms.

### Muscle biopsies and storage

Biopsy specimens were obtained at the beginning of hip replacement surgery under general anaesthesia. The edge of the tensor fascia femoris was exposed and a 1 cm^3^ muscle specimen was removed using sharp dissection. Then, small bleeding vessels were carefully coagulated by high-frequency electrosurgical equipment and the operation continued in routine fashion. No complications occurred from the biopsy procedure. After removing excess fat and connective tissue, the biopsy specimens were flushed with cold PBS to rinse the blood. The muscle specimens were immediately frozen in liquid nitrogen and stored at -80 °C until further analysis.

### Histological and ultrastructural analyses

Haematoxylin and eosin (H&E) staining and transmission electron microscopy (TEM) observation of the tensor fascia femoris muscle were performed following standard protocols. For H&E staining, biopsy specimens were fixed in 4% paraformaldehyde, embedded in paraffin, and then cut into sections. After staining with H&E, the sections were visualized under a light microscope system (Leica, Germany). Images of muscle section were taken randomly from sample, and the average cross-section areas (CSA) of muscle fibre was determined by manually encircling each cross-section of at least 100 fibres using standard imaging software ImageJ (v1.6.0, NIH, Bethesda, USA) (*n* = 6 per group). For ultrastructural analyses, the fixation and Epon embedding of ultrathin sections were performed according to standard procedures, and the morphology of the biopsy specimens was observed using a transmission electron microscope (Hitachi, Japan) at an acceleration voltage of 80 kV. Three randomly selected fields within a 32.6 μm2 field of view for each sample were analysed to assess the ultrastructure of myofibrils, sarcomeres, lipid droplets, and mitochondria.

### RNA Extraction, Reverse Transcription, and RT‒qPCR

RT‒qPCR was conducted according to standard methods on SP and NSP. Briefly, total RNA was extracted from frozen muscle biopsies with Trizol reagent (Invitrogen Life Technologies, USA), and the RNA quantity was determined by NanoDrop2000 (Life Technologiess, USA). All RNA samples were homogeneous and passed quality control with 260/280 nm ratio > 1.8. Then cDNA of each sample was synthesized using 1.0 µg of RNA and the PrimeScript RT Reagent Kit (RR037A, Takara, Japan) according to the manufacturer’s instructions. The qPCR was performed by QuantStudioTM Dx real-time PCR instrument (ThermoFisher Scientific, Singapore) using TB Green Premix ExTaq II (RR820A, Takara, Japan) according to the manufacturer’s instructions. The relative gene expression was calculated using the ΔΔCt method, and GAPDH was used as the internal control. The primers used in this q-PCR reactions were listed in Additional file 1.

### Statistical analyses

For descriptive statistics, the continuous variables with a normal distribution were expressed as the mean ± standard deviation (SD), while the median (range) was used for an abnormal distribution. Classified variables were expressed by counts and percentages. Only two-tailed *p* < 0.05 was considered statistically significant. All bioinformatics statistical analysis was performed using R version 3.5.3 (Institute for Statistics and Mathematics, Vienna, Austria; https://www.r-project.org).

## Results

### Identification of DEGs and functional enrichment analysis

**Fig. 1:**
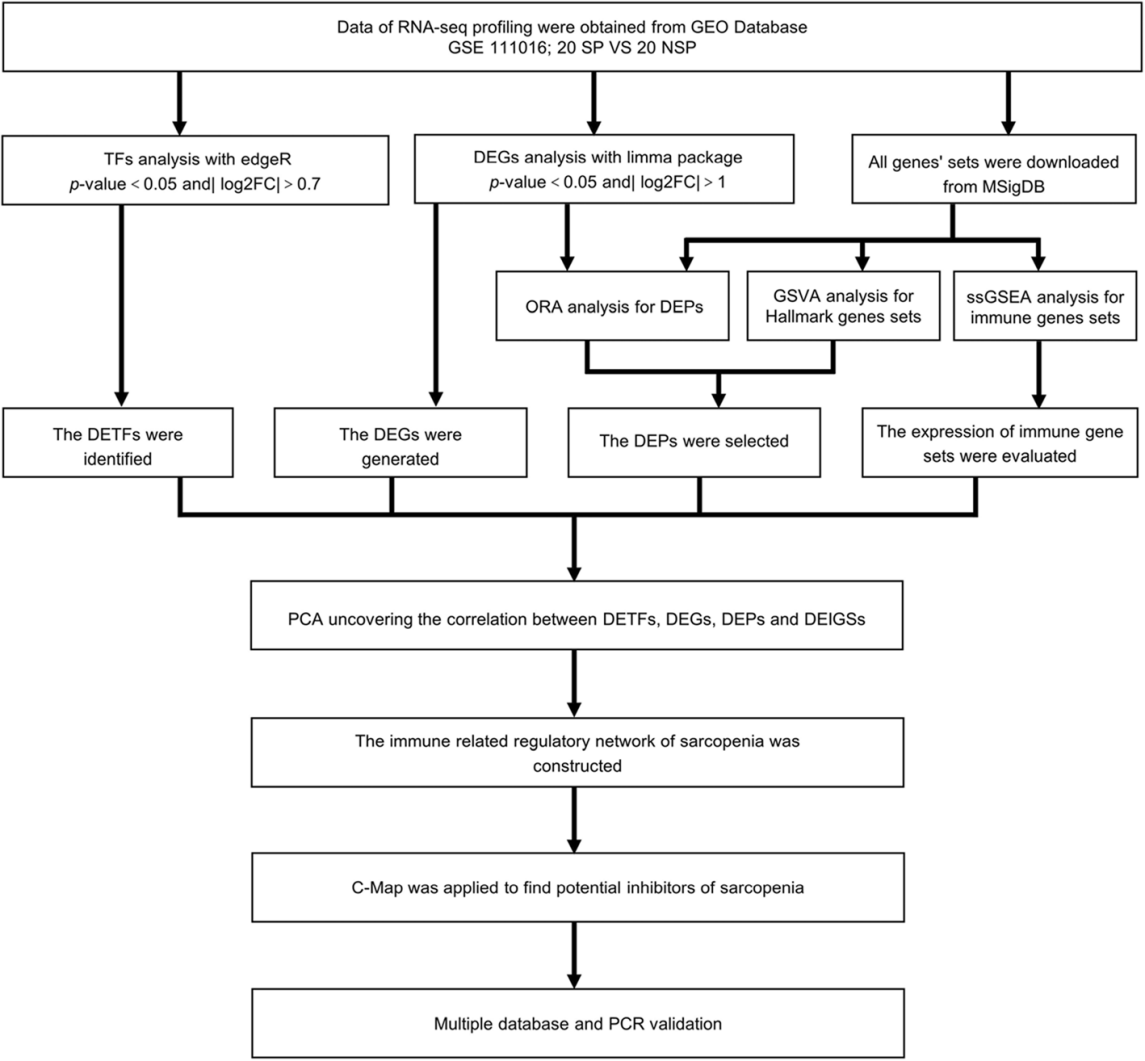
The flow chart of this study

**Fig. 2:**
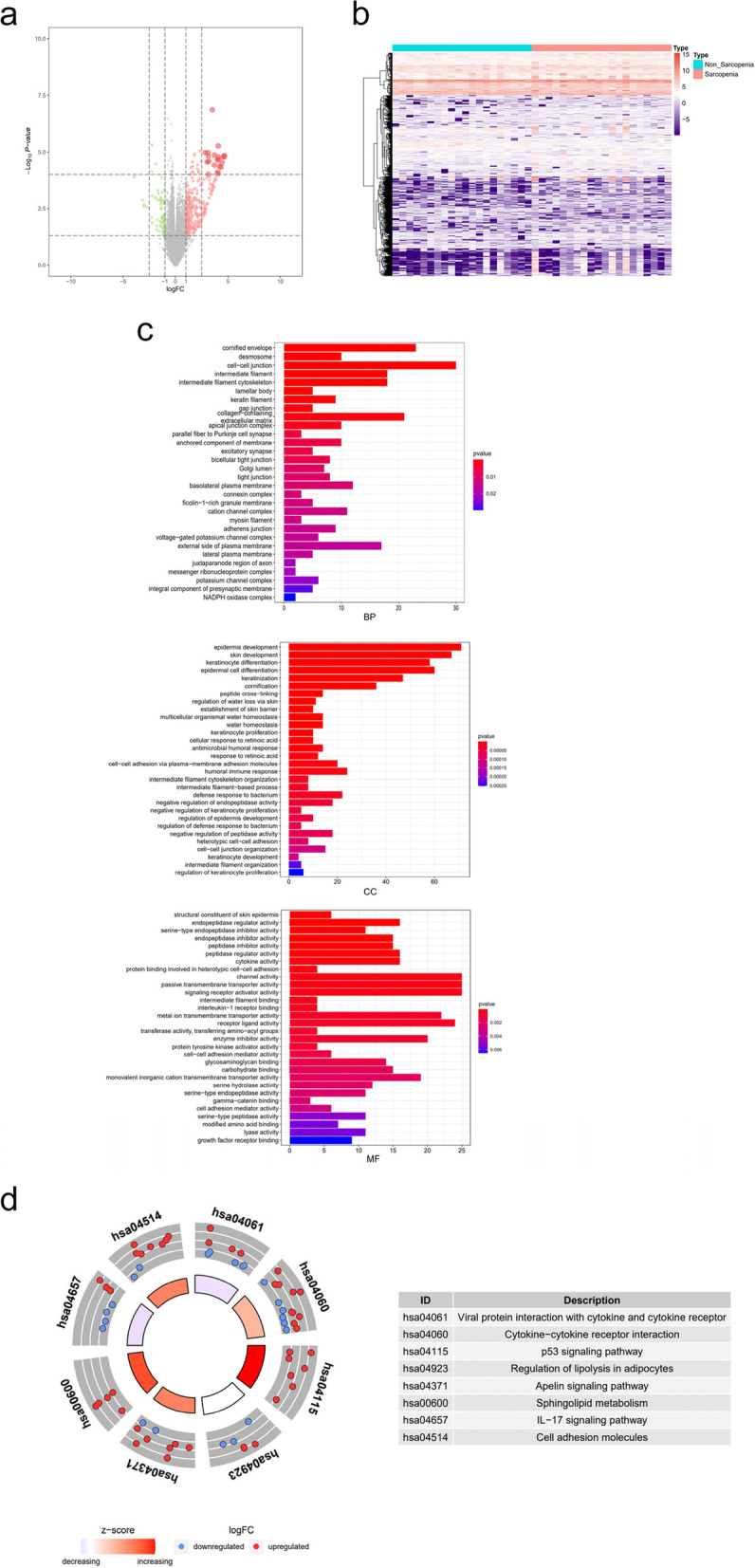
The DEGs and Functional enrichment analysis. **a** The volcano plot and (**b**) heatmap of 808 DEGs between 20 SP and 20 NSP. **c** The GO terms of BP, CC, MF, and (**d**) KEGG analyses of 808 DEGs. The “Epidermis development” for BP (GeneRatio = 0.163, *p* < 0.0001, count = 71), “cornified envelope” for CC (GeneRatio = 0.0513, *p* < 0.0001, count = 23) and “structural constituent of skin epidermis” for MF (GeneRatio = 0.014, *p* < 0.0001, count = 6) were the most significant GO items. The “Viral protein interaction with cytokine and cytokine” (GeneRatio = 0.0497, *p* = 0.0003, count = 9) was the most significant KEGG item

The bioinformatics analysis flow chart of this study is illustrated in Fig. 1. The patients baseline features of each muscle sample were also collected from the GEO database and are described in Additional file 2. The genes with a **|**log2FC**|**> 1 and *p* < 0.05 between SP and NSP were defined as DEGs, and we identified 808 DEGs (207 downregulated and 601 upregulated) (Additional file 3), which is illustrated by a volcano plot and heatmap (Fig. 2a, 2b). To examine the different biological phenotypes and the potential mechanisms of the DEGs, GO and KEGG enrichment analyses were performed. “Epidermis development” for BP (GeneRatio = 0.163, *p* < 0.0001, count = 71), “cornified envelope” for CC (GeneRatio = 0.0513, *p* < 0.0001, count = 23) and “structural constituent of skin epidermis” for MF (GeneRatio = 0.014, *p* < 0.0001, count = 6) were the most significant GO items (Fig. 2c). KEGG enrichment analysis showed that “Viral protein interaction with cytokine and cytokine” (GeneRatio = 0.0497,* p* = 0.0003, count = 9) was the most significant item (Fig. 2d). In addition, several immune response processes, such as “humoral immune response”, “cytokine activity”, “interleukin-1 receptor binding”, “IL-17 signalling pathway” were also significantly different in GO analysis and KEGG analysis (Additional file 4), indicating that immune-related mechanisms were involved in sarcopenia.

### Identification of potential signalling pathways in sarcopenia

**Fig. 3:**
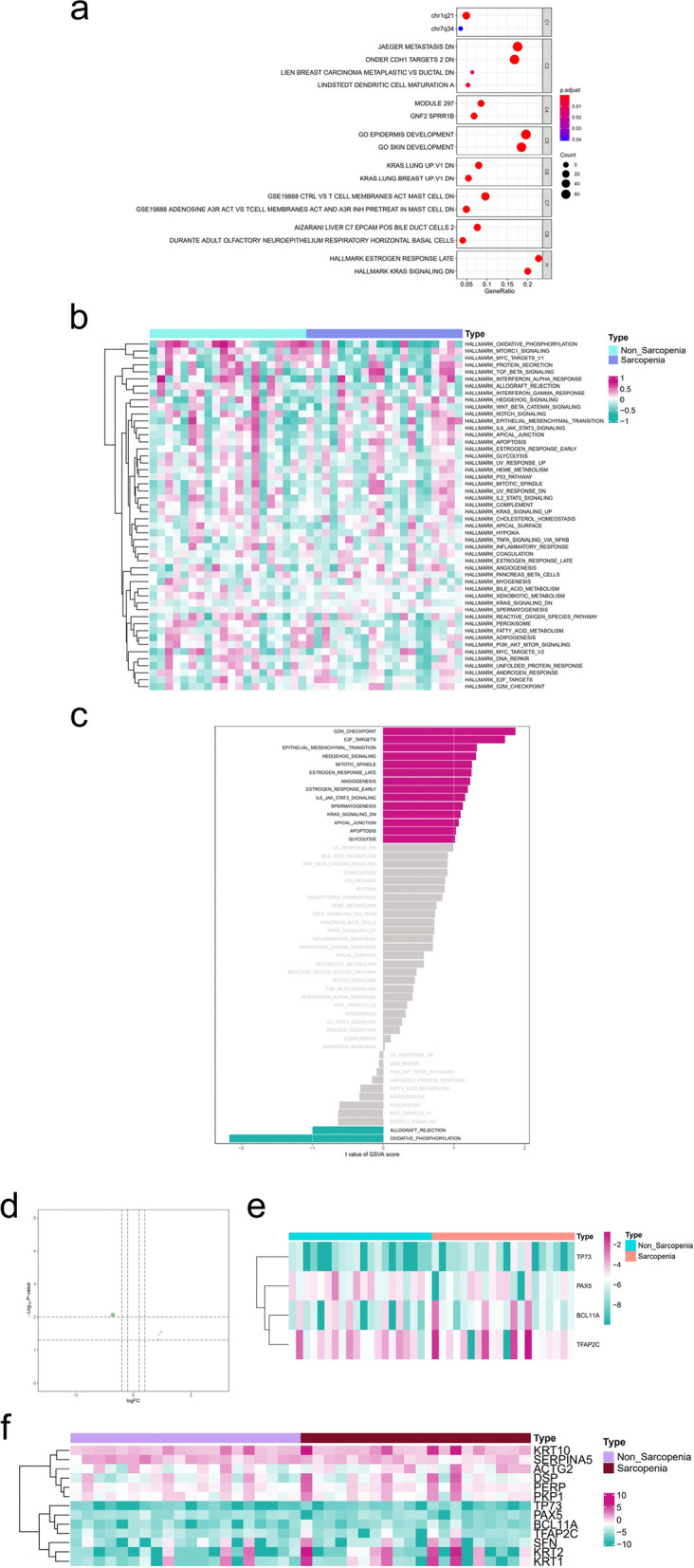
Identification of potential signaling pathways, DETFs and final DEGs in sarcopenia. **a** The ORA analysis of DEPs, and the bubble plot represents the top 2 or 4 differentially expressed genes sets for each collection. **b** The heatmap and (**c**) result of GSVA analysis of hallmark gene sets. The histogram showed 14 up-(red bar) and 2 down-regulated (green bar) differentially expressed sarcopenia-related pathways. **d** The volcano plot and (**e**) heatmap of 4 DETFs between 20 SP and 20 NSP. The expressional levels of TP73, BCL11A and TFAP2C were significantly increased in sarcopenia, while the PAX5 exhibited significantly decreased. **f** The heatmap of DETFs and final DEGs according to the result of PCA

To uncover the deeper mechanism underlying sarcopenia, ORA enrichment analysis was performed. We explored the alteration of sarcopenia in 8 major collections of MSigDB and analysed the differentially expressed gene sets systematically between the NSP and SP. By using ORA, we identified 288 differentially expressed gene sets (Additional file 5) and the bubble plot represents the top 2 or 4 differentially expressed gene sets for each collection (Fig. 3a). Furthermore, many immune related gene sets, such as “ZHOU_INFLAMMATORY_RESPONSE_LPS_UP” (GeneRatio = 0.0303, *p* < 0.0001) in C2, and “GO_ANTIMICROBIAL_HUMORAL_RESPONSE” (GeneRatio = 0.0167, *p* < 0.0001) in C5, were also significantly upregulated in sarcopenia (Additional file 5).

To avoid the disadvantages of artificially setting thresholds of DEGs in KEGG and ORA, GSVA was also performed to explore the significantly altered hallmark pathways, and the expression levels of gene sets for each sample are shown in the heatmap (Fig. 3b). Through GSVA, we identified 16 DEPs, including 14 upregulated and 2 downregulated pathways (Fig. 3c). “OXIDATIVE_PHOSPHORYLATION” (ES = 2.1680) and “G2M_CHECKPOINT” (ES =  − 1.8655) were the most downregulated and upregulated in sarcopenia, respectively. In addition, the levels of the “ESTROGEN_RESPONSE”, “APOPTOSIS” and “GLYCOLYSIS” were also significantly elevated in sarcopenia (Fig. 3c).

### Identification of DETFs, final DEGs and final DEPs in sarcopenia

Sequence data for 318 TFs were retrieved from the Cistrome database. The DETFs were screened with *p* < 0.05 and |log2FC|> 0.7 by using the edgeR method (Fig. 3d). We identified 4 DETFs, including 3 upregulated and 1 downregulated, and the expression of DETFs in the two groups is displayed in the heatmap in Fig. 3e. Among the four DETFs, only PAX5 exhibited significantly decreased expression, while TP73, BCL11A and TFAP2C were increased in sarcopenia. Then, the PCA was performed to screen the DETFs-related fianl DEGs, and the final DEGs were defined as those having absolute values of PCC > 0.4 and *p* < 0.01. We identified 9 final DEGs, including 1 downregulated and 8 upregulated, and the results of DETFs and final DEGs are illustrated by the heatmap in Fig. 3f.

Next, the correlation between final DEGs and DEPs was performed by PCC, and the DEPs with an absolute value of PCC > 0.4 and *p* < 0.01 were retained for further regulatory network analysis. As a result, 11 final DEPs were included, and the immune-related pathways “INFLAMMATORY_RESPONSE”; metabolic abnormalities-related pathways “PI3K_AKT_MTOR_SIGNALING”, “XENOBIOTIC_METABOLISM”, and “HYPOXIA”; and apoptosis-related pathway “APOPTOSIS” were the most relevant among those 11 finals DEPs (Additional file 6).

### Construction of a novel transcriptional regulatory network with an immune signature in sarcopenia

**Fig. 4:**
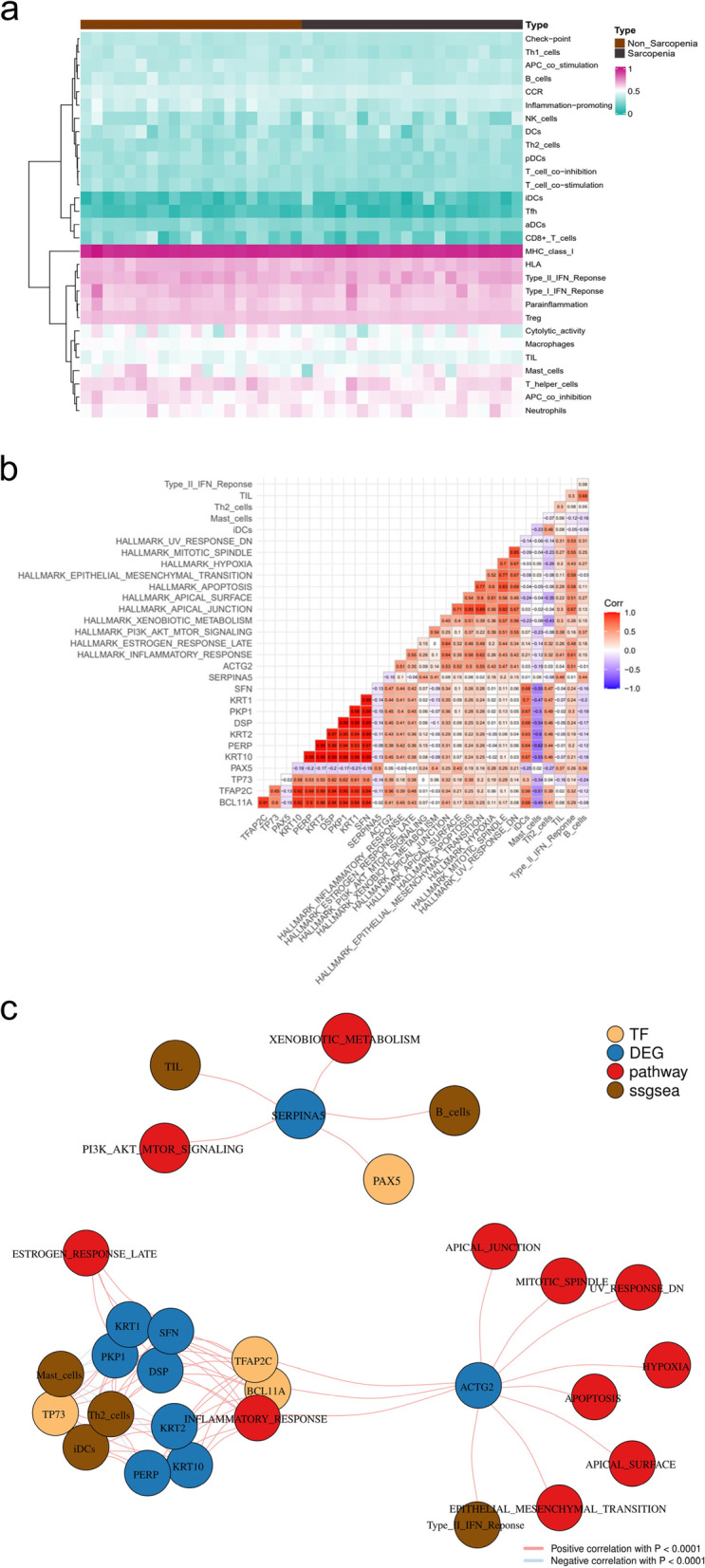
Identification of DEIGSs in sarcopenia and the construction of immune-related transcriptional regulatory network. **a** The heatmap of immune gene sets of 40 samples by ssGSEA. **b** The co-expression heatmap and (**c**) network plot of DETFs, final DEGs, final DEPs and final DEIGSs

To determine the immune-related transcriptional regulatory network in skeletal muscle with sarcopenia, the ssGSEA algorithm was applied to filter significant immune cells and pathways. First, we analysed 29 immune-associated gene sets for each sample (Additional file 7), and the result are displayed by the heatmap in Fig. 4a. Then, PCA was applied to identify the correlation between the DEGs and immune cells and pathways, and the DEIGSs with absolute values of PCC > 0.4 and *p* < 0.0106 were included in the subsequent analysis. After the screening procedure described above, we obtained 1208 DEIGSs and 459 corresponding DEGs (Additional file 8). The final DEIGSs were further identified by backtracking the corresponding DEIGSs of the final DEGs. Finally, we identified 6 DEIGSs in sarcopenia, including 5 types of immune cells and 1 immune response. The 6 final DEIGSs correlated with sarcopenia were iDCs (CC = 0.698, *p* < 0.001, positive), mast cells (CC =  − 0.621, *p* < 0.001, negative), Th2 cells (CC = 0.488, *p* = 0.001, positive), TIL (CC = 0.460, *p* = 0.003, positive), B cells (CC = 0.437, *p* = 0.005, positive) and type II IFN response (CC = 0.511, *p* < 0.001, positive). Finally, the coanalysis for DETFs, final DEGs, final DEPs and final DEIGSs was performed(Additional file 9), and the result is shown by the coexpression heatmap in Fig. 4b. To display our results more clearly, a network was plotted by Cytoscape 3.7.1. Finally, the sarcopenia-related hypothesis built on bioinformatics is displayed in a s signalling diagram in Fig. 4c.

### Identification of specific inhibitors by C-Map

**Fig. 5:**
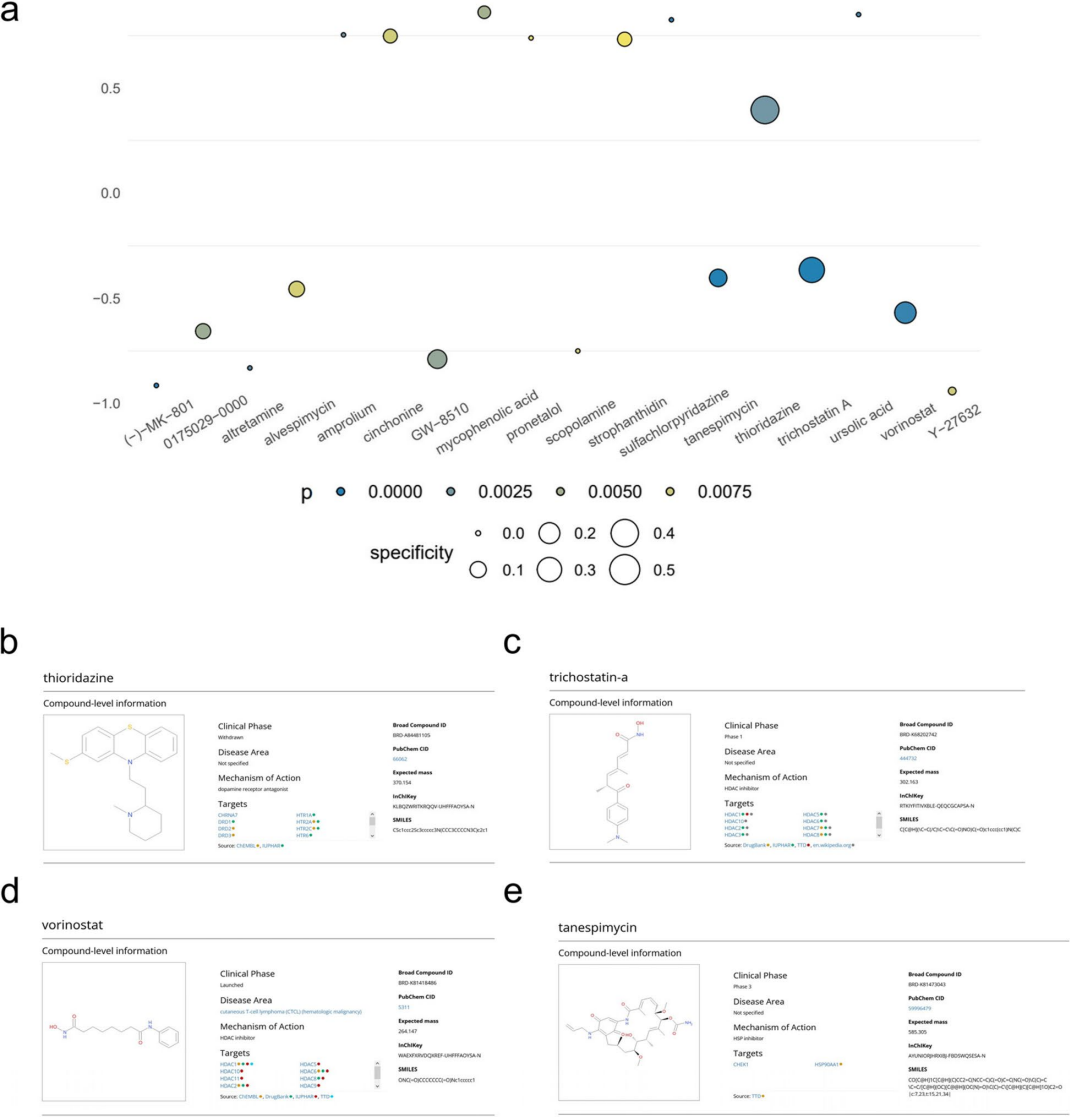
**a** The C-Map of sarcopenia and (**b**-**e**) detail information of inhibitors. The information of (**b**) Thioridazine, (**c**) Trichostatin A, (**d**) Vorinostat and (**e**) Tanespimycin from Pubchem database

To provided potential treatment strategy for sarcopenia, C-Map was utilized to identify potential inhibitors of the network. We screened 1309 revealed bioactive compounds in the C-Map database (Additional file 10), and 18 potential specific inhibitors targeting the network were identified with* p* < 0.01 (Fig. 5a). Among those compounds, “tanespimycin” (enrichment =  − 0.403, specificity = 0.1681, *p* < 0.0001), “trichostatin A” (enrichment =  − 0.365, specificity = 0.4257, *p* < 0.0001), “vorinostat” (enrichment =  − 0.568, specificity = 0.4257, *p* = 0.00038), and “thioridazine” (enrichment = 0.396, specificity = 0.5388, *p* = 0.00242) had the most potential as inhibitors of sarcopenia according to the results of C-Map. Based on the PubChem database, detailed information of thioridazine (Fig. 5b), trichostatin A (Fig. 5c), vorinostat (Fig. 5d) and tanespimycin (Fig. 5e) were further confirmed. Finally, the compound of trichostatin A was the most promising potential medicine for sarcopenia according to the results of the literature review and C-Map [14–16].

### Clinical characterization of patients with and without sarcopenia

To support our hypothesis, histological and morphological analyses of skeletal muscle between SP and NSP were performed. The biopsy specimens were obtained from a prospective cohort study, 12 participants were diagnosed with sarcopenia, and 12 were nonsarcopenia according to the 2019 AWGS (Additional file 8). Muscle mass and function were significantly different as grip strength, lean body mass and ALMi for NSP and SP were 34.2 ± 3.6 kg vs. 23.0 ± 2.8 kg, 43.5 ± 2.7 kg vs. 38.8 ± 1.6 kg, and 7.6 ± 0.3 kg/m^2^ vs. 6.3 ± 0.3 kg/m^2^, respectively. The laboratory test results revealed that the sarcopenia patients had higher levels of inflammation and worse function reserve, as the C-reaction protein, leukocyte, haemoglobin, and albumin levels for NSP and SP were 24.7 ± 7.0 g/dL vs. 37.2 ± 10.7 g/dL, 6.8 ± 1.2* 10^9^/L vs. 9.0 ± 1.9*10^9^ /L, 137.0 ± 10.5 g/L vs. 122.7 ± 9.9 g/L, 41.8 ± 2.3*10^9^ /L vs. 38.9 ± 1.8*10^9^ /L, respectively. There was no difference in age, BMI, NRS-2002 score, platelets, or fat mass content between the two groups, and all patients’ demographic information is summarized in Table 1.

**Table 1:** Clinical characteristics of patients with or without sarcopenia

**Variables**	**Non-sarcopenia** (*n* = 12)	**Sarcopenia** (*n* = 12)	***p*-value** (Control vs Sarcopenia)
Age (years,Mean ± SD)	74.8 ± 4.7	75. 4 ± 4.1	N.S
BMI (kg/m^2^,Mean ± SD)	21.9 ± 1.4	21.8 ± 1.9	N.S
Grip strength (kg,Mean ± SD)	34.2 ± 3.6	23.0 ± 2.8	< 0.001
Lean body mass(kg,Mean ± SD)	43.5 ± 2.7	38.8 ± 1.6	< 0.001
Fat Mass(kg,Mean ± SD)	15.5 ± 1.1	15.9 ± 1.1	N.S
ALMi(kg/m^2^,Mean ± SD)	7.6 ± 0.3	6.3 ± 0.3	< 0.001
NRS2002 score(Media,IQR)	2,1.0	2,1.8	
History of tobacco use(n)	3	4	
History of alcohol use(n)	2	3	
Drugs (n)			
Antihypertensive drugs	2	2	
Antihyperglycemic drugs	1	2	
Antihyperlipidemic drugs	1	1	
Laboratory data			
C-reaction protein(g/dL)	24.3 ± 7.0	37.2 ± 10.7	0.002
Leukocyte(10^9 /L)	6.8 ± 1.2	9.0 ± 1.9	0.003
Hemoglobin(g/L)	137.0 ± 10.5	122.7 ± 9.9	0.002
Platelets(10^9 /L)	195.8 ± 68.5	222.2 ± 71.5	N.S
Albumin(g/L)	41.8 ± 2.3	38.9 ± 1.8	0.003

### Identification of muscle fibre size and ultrastructural changes in sarcopenia

**Fig. 6:**
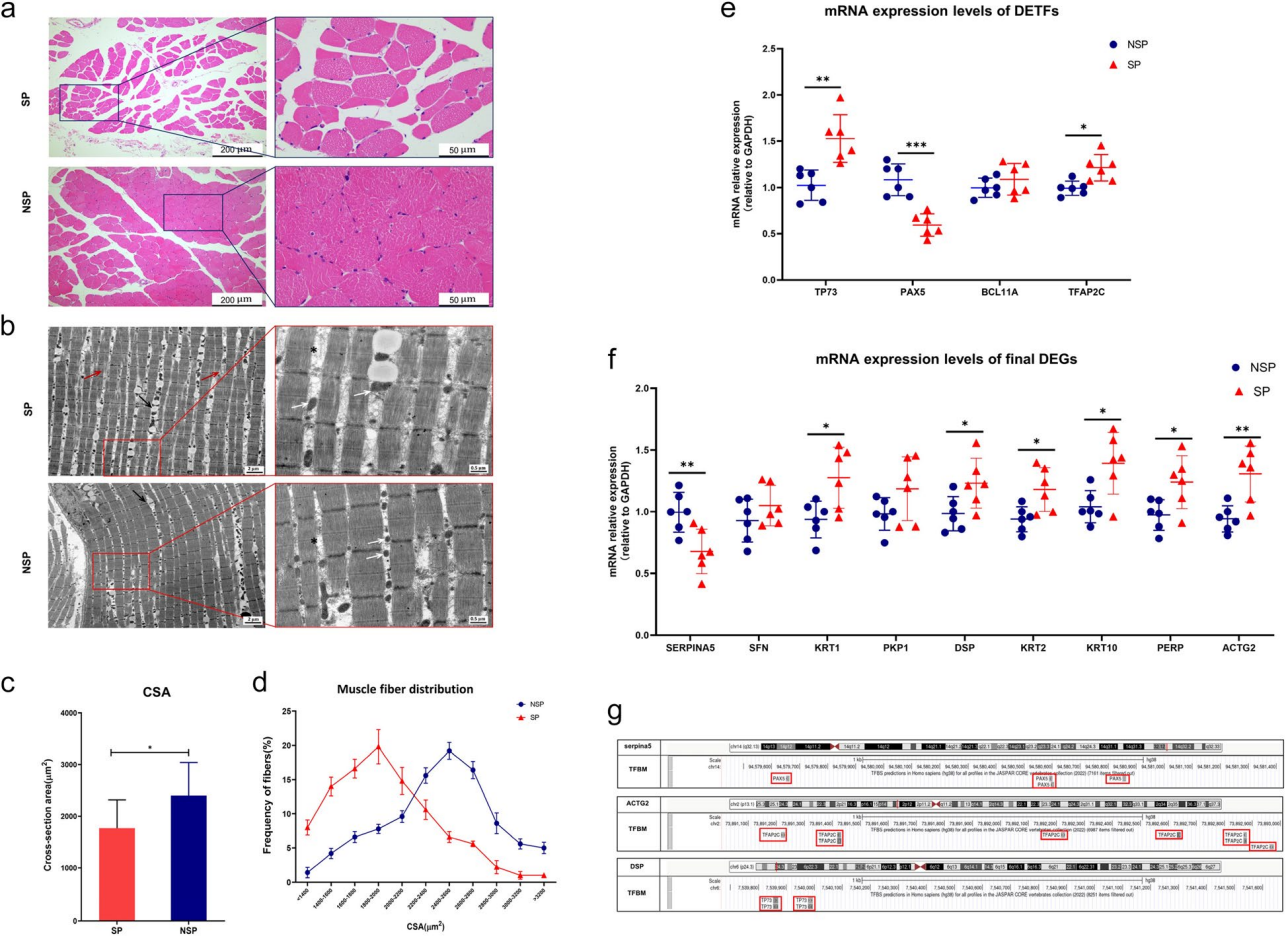
Identification of muscle morphology change in sarcopenia and validation of the regulatory mechanism between the DETFs and final DEGs. **a** Representative images of the tensor fascia femoris muscle fiber by H&E staining. Muscle fiber atrophy and the decrease of CSA was observed in SP **c**, **d**. (**b**) Representative images of TEM from the SP and NSP. In SP, myofibrils appeared atrophied and the interspace between myofibrils were enlarged (black asterisk). Myofilaments and sarcomeres became ruptured, fused, or disappeared (dark red arrows) and the number of lipid droplets (black arrows) between myofibrils significantly increased in SP. The percentage of abnormal mitochondria (white arrows) was also significantly increased in the SP. **e** The mRNA expressional levels of DETFs showed that TP73, TFAP2C were significantly upregulated in SP and the PAX5 was obviously downregulated in SP, while the BCL11A had no significant change between SP and NSP. **f** The expression of KRT10, ACTG2, DSP, PERP, KRT2, KRT1, BCL11A and TFAP2C, increased significantly, while the expression level of SERPINA5, which might be positive regulated by PAX5, decreased significantly. **g** The TFBM of PAX5, TFAP2C and TP73 were identified located in promoters of SERPINA5, ACTG2 and DSP respectively by using NCBI, UCSC and JASPAR database

The representative images of H&E demonstrated significant changes in the diameter of muscle fibres between the two groups (Fig. 6a). In cross-sections, muscle fibre atrophy was observed, and the CSA was significantly decreased in SP (1855 ± 499μm2) when compared with the NSP (2402 ± 604μm2) (Fig. 6c). As compared with NSP, the distribution of CSA was also significantly reduced in SP(Fig. 6d). TEM further revealed the aberrant ultrastructural changes of myofibrils (Fig. 6b). In NSP, the myofibrils were plump, well-organized, and attached closely with each other. However, in SP, myofibrils appeared mildly atrophied and the interspace between myofibrils (black asterisk) was enlarged. Many myofilaments and sarcomeres became ruptured, fused, or disappeared (dark red arrows) in SP, and the number of lipid droplets (black arrows) between myofibrils also significantly increased. In addition, the size and shape of mitochondria varied greatly, and the percentage of abnormal mitochondria (white arrows) was also significantly increased in SP. All these anomalies in the quantity, morphology, and distribution of myofibrils, mitochondria, and lipid droplets indicated the occurrence of muscle dysfunction in SP. For more representative images of TEM, please see the Additional file 11.

### Identification of DETFs and final DEGs in the study population by RT‒qPCR

To repredict the transcriptional regulatory pattern of DETFs and final DEGs, the RNA expression levels of biopsies were detected by RT‒qPCR. The RNA expression results of 4 DETFs showed that TP73 and TFAP2C were significantly upregulated, and PAX5 was obviously downregulated in SP, while BCL11A had no significant change between the two groups (Fig. 6e). Then, we tested the RNA expression levels of the 9 final DEGs. As shown in Fig. 6f, the expression levels of KRT10, ACTG2, DSP, PERP, KRT2 and KRT1, which are associated with TP73, BCL11A and TFAP2C, increased significantly, while the expression level of SERPINA5, which might be positively regulated by PAX5, was significantly decreased. At the same time, the expression levels of PKP1 and SFN, might be positive regulated by P73, BCL11A and TFAP2C, and showed no significant changes between SP and NSP.

### External validation of the regulatory mechanism with multiple online databases

For further validation of the regulatory mechanism between the DETFs and final DEGs, a comprehensive retrieval of public databases was performed to search for the presence of putative DETF binding sites located within the transcriptional regulatory region of the final DEGs. First, we found the promoters of the final DEGs with NCBI and ensured that the potential direct transcription factor binding motifs (TFBM) of PAX5, TFAP2C and TP73 were located in the promoters of SERPINA5, ACTG2 and DSP respectively, and the results are illustrated in Fig. 6g. Next, JASPAR in-silico analysis was performed to determine the binding site sequences of DETFs. The precise binding sites and the relative score are illustrated in Table 2 for DETFs with a relative profile score threshold higher than 80%. In the promoter regions of SERPINA5, ACTG2 and DSP, 4 putative binding sites for PAX5 and TP73, and 5 putative binding sites for TFAP2C were detected with relative score ranging from 0.80 to 0.85. All the final DEGs reported in Table 2 show more than one putative binding site for DETFs, thus suggesting that all of them may be potentially bound by DETFs within their regulatory region.

**Table 2:** JASPAR analysis results for DETFs binding sites within the promoter of key DEGs

DEG Name	TF Matrix ID	TF Name	Score	Relative score	Sequence ID	Start	End	Strand	Predicted sequence
Serpina5	MA0014.3	MA0014.3.PAX5	8.354057	0.835880295	NC_000014.9:94579426–94581426	1427	1438	+	ATGCATGACTTT
	MA0014.3	MA0014.3.PAX5	7.273882	0.813180180	NC_000014.9:94579426–94581426	171	1182	-	GTGCATGGCCGT
	MA0014.3	MA0014.3.PAX5	6.896367	0.805246625	NC_000014.9:94579,426–94581426	228	239	-	ATCCGTGTCCTA
	MA0014.4	MA0014.3.PAX6	6.66627	0.800411082	NC_000014.9:94579426–94581426	115	1176	-	GGCCGTGATCAT
ACTG2	MA0815.1	MA0815.1.TFAP2C	6.9714537	0.833060697	NC_000002.12:73891008–73893008	1861	1873	+	CGCCTTGGGGGTA
	MA0815.1	MA0815.1.TFAP2C	6.731798	0.829739041	NC_000002.12:73891008–73893008	1861	1873	-	TACCCCCAAGGCG
	MA0815.1	MA0815.1.TFAP2C	5.5529466	0.813400009	NC_000002.12:73891008–73893008	1601	1613	+	AGACCCAGGGGCC
	MA0815.1	MA0815.1.TFAP2C	5.440579	0.811842579	NC_000002.12:73891008–73893008	1601	1613	-	GGCCCCTGGGTCT
	MA0815.1	MA0815.1.TFAP2C	4.90462	0.804414123	NC_000002.12:73891008–73893008	1862	1874	-	TCGCCTTGGGGGT
DSP	MA0861.1	MA0861.1.TP73	10.096636	0.850966062	NC_000006.12:7539671–7541671	1861	1873	+	CGCCTTGGGGGTA
	MA0861.1	MA0861.1.TP73	8.66093	0.835814809	NC_000006.12:7539671–7541671	1861	1873	-	TACCCCCAAGGCG
	MA0861.1	MA0861.1.TP73	8.087384	0.829762085	NC_000006.12:7539671–7541671	1601	1613	+	AGACCCAGGGGCC
	MA0861.1	MA0861.1.TP73	6.944413	0.817700116	NC_000006.12:7539671–7541671	1862	1874	-	TCGCCTTGGGGGT

## Discussion

As a chronic common disease among the elderly population (present in 11–50% of people aged > 80 years) [17], sarcopenia was officially recognized as a muscle disease and awarded the ICD-10-CM (M62.84) code in 2016 [18]. As the global population ages, the incidence rate of sarcopenia is rapidly increasing [19], which increases the medical cost burden on society. The coexistence of sarcopenia with other diseases also complicates the diagnosis and treatment of patients’ diseases [20]. However, the related mechanisms of sarcopenia have not yet been clearly explored, and there are no specific treatment drugs [3].

In this study, we primary screened 808 DEGs, 4 DETFs, 304 DEPs and 1208 differential expressed immune cell- or immune response-related events. Based on the results of PCA, we then structured an immune-related transcription factor regulatory network with 4 DETFs, 9 final DEGs, 11 final DEPs, 5 immune cells and 1 immune reaction. Combining the identification results of online databases and in vitro experiments, we speculated that PAX5-SERPINA5-PI3K/Akt axis was the most potential pathogenic pathway and that B cells may play a vital role in signal transduction. Finally, according to the results of the literature review and C-Map, we hypothesized that trichostatin A might be a potential medicine for sarcopenia.

TFs can direct genome expression and alter the activity of the signalling pathway by recognizing specific DNA sequences and controlling chromatin transcription to form RNA [5]. With the increasing available information about TFs and their target genes, it has become more convenient for us to study the transcriptional regulatory network with multiple methods and databases. Recently, many studies have reported that both cellular and molecular features-related TFs exert important influences on the anabolism and catabolism of proteins in muscle, and many TFs have been proven to play a crucial role in the regulation of sarcopenia [9]. For example, Nrf2 and FoxO3a are important TFs that are responsible for the progression of cellular protein homeostasis and carbohydrate storage. They can regulate the metabolic homeostasis of muscle fibres by affecting the pathways involved in oxidative stress, cell metabolism, apoptosis, and so on [21, 22]. In this study, we investigated the relationship between the PAX5-SERPINA5-PI3K/Akt axis and sarcopenia for the first time. Consistent with the literature, we found that PAX5 and SERPINA5 were positively correlated with muscle function and that their expression levels were significantly reduced in sarcopenia [23, 24]. For further validation of the regulatory mechanisms between PAX5 and SERPINA5, we confirmed the potential transcription factor binding motif and binding sites by using the NCBI, UCSC and JASPAR databases.

PAX5, also known as a paired box 5, was the only downregulated transcription factor among the 4 DETFs in our study. PAX5 encodes a member of the PAX family and is a vital regulator of cell development, tissue differentiation and immune function [25]. Previous studies have revealed that PAX5 promotes the proliferation of muscle satellite cells and regeneration of muscle fibres in mice [20]. Shoji Fukuda et al. demonstrated that PAX5 might have a positive effect on improving myocardial function by inhibiting the apoptosis of cardiomyocytes and endothelial cells, as well as increasing microvascular density in a chronic rat myocardial infarction model [26]. PAX5 can also inhibit inflammatory and immune responses by altering the chromatin transcriptional activity of key genes, such as NF-κB, TLR4, FOS, and AKT3 [27, 28]. Since it has also been proposed to have a major role in the development and renewal of neurons, PAX5 may also have a close relationship with neuromuscular junction disorders in sarcopenia [29]. The latest study found that PAX5 might also play an important role in the metabolism of skeletal muscle tissue by activating the transcription of ITGB3 [30].

As one of the extracellular alpha-1-antitrypsin clades of serpin, SERPINA5 can inhibit several plasma serine proteases and plays diverse roles in host defence, protein haemostasis, and lipid transport in multiple organs [31]. Previous studies have found that SERPINA5 could suppress inflammation by inhibiting the activation of protein C, which has a proinflammatory function by affecting blood coagulation [32]. Rajeevan et al. found that genetic variants of SERPINA5 were associated with alterations in inflammation and immune pathways in elderly individuals [33]. A clinical study also demonstrated that SERPINA5 is associated with physical function in genetic chronic fatigue syndrome [24]. In addition, aberrantly expressed SERPINA5 contributed to heart failure and osteoarthritis in the elderly [34]. SERPINA5 also contributes to the regulation of many important cellular functions, such as regeneration, survival, and cell death by regulating intracellular MAPK and lipid signalling pathways [35].

Several recent studies have shown that immune cells play an important role in the development and progression of sarcopenia [8]. However, the relationship between the immune system and sarcopenia has not been comprehensively assessed by high-throughput analysis. As sarcopenia shows a close relationship with cancer, inflammation, and immunity, we also explored the alteration in 26 immune gene sets by ssGSEA [36]. Combining the identification results of online databases, we found that lymphocytes may play a vital role in the development of sarcopenia. As an indispensable component of the humoral adaptive immune system, B cells play an important role against a variety of pathogens by infiltrating into tissues and secreting antibodies [37]. Defects in B-cell function lead to many immune diseases, such as inflammation [38]. Previous studies proved that the fate of cells is largely regulated by cytokines and TFs, and PAX5 could play an essential role in controlling the development and function of B cells [39]. In accordance with our results, the latest study demonstrated that PAX5 could regulate B-cell humoral immunity by promoting PI3K signalling [40]. Here, we first discovered that the PAX5-SERPIAN5-PI3K/Akt axis was a potential mechanism in muscle dysfunction, and B cells may play a vital role in this signal transduction. Our findings also provide novel predictors and therapeutic targets for sarcopenia and indicate that trichostatin A might be a useful drug for sarcopenia.

Several limitations in our study should be noted. First, the muscle RNA-seq data in our analysis were collected from public databases, and much clinical information was incomplete, such as basic illness and medication use, which may cause potential bias and even errors. Second, the network demonstrated in our study was based on bioinformatics analysis and multidimensional correlation rather than biological mechanism research. Therefore, more cell and animal experiments still need to be performed to support our findings. In the future, we will conduct functional experiments to verify the PAX5-SERPINA5-PI3K/Akt axis in vivo and in vitro.

## Conclusion

In conclusion, our study was the first to predict the immune-related transcriptional regulatory network with high-throughput RNA-seq data in sarcopenia. We supposed that PAX5-SERPIAN5-PI3K/Akt axis is a potential mechanism in sarcopenia, and B cells may also play a vital role in this signal transduction. In addition, trichostatin A might be a potential medicine for sarcopenia.

## Supplementary Information

**Additional file 1.** Primers of DETFs and final DEGs.

**Additional file 2.** Results of DEGs.

**Additional file 3.** Functional enrichment analysis.

**Additional file 4.** Results of ORA.

**Additional file 5.** Results of DEPs.

**Additional file 6.** Results of ssGSEA.

**Additional file 7.** Results of DEIGSs.

**Additional file 8.** Results of co-analysis.

**Additional file 9.** Results of C-Map database.

**Additional file 10.** Clinical characterization of patients.

**Additional file 11.** The representative images of TEM.

## Data Availability

The datasets used and analysed during this study are available from the corresponding author on reasonable request.

## Abbreviations

DEGs: Differentially expressed genes
FC: Fold change
DETFs: Differentially expressed transcription factors
DEPs: Differentially expressed pathways
ES: Enrichment scores
CC: Correlation coefficient
TFs: Transcription factors
GEO: Gene Expression Omnibus
GO: Gene Ontology
KEGG: Kyoto Encyclopedia of Genes and Genomes
ORA: Overrepresentation analysis
GSVA: Gene set variation analysis
ssGSEA: Single-sample gene set enrichment analysis
C-Map: Connectivity Map
AWGS: Asian Working Group for Sarcopenia Consensus
SP: Sarcopenia group
NSP: Nonsarcopenia group
DSM-BIA: Direct segmental multifrequency bioelectrical impedance analysis
CSA: Cross-section area
