# Supplementary figures and images for: Identification of a novel immune-related transcriptional regulatory network in sarcopenia
Source: BMC Geriatr. 2023 Jul 31;23:463. doi: 10.1186/s12877-023-04152-1 (PMC10391869; doi:10.1186/s12877-023-04152-1)

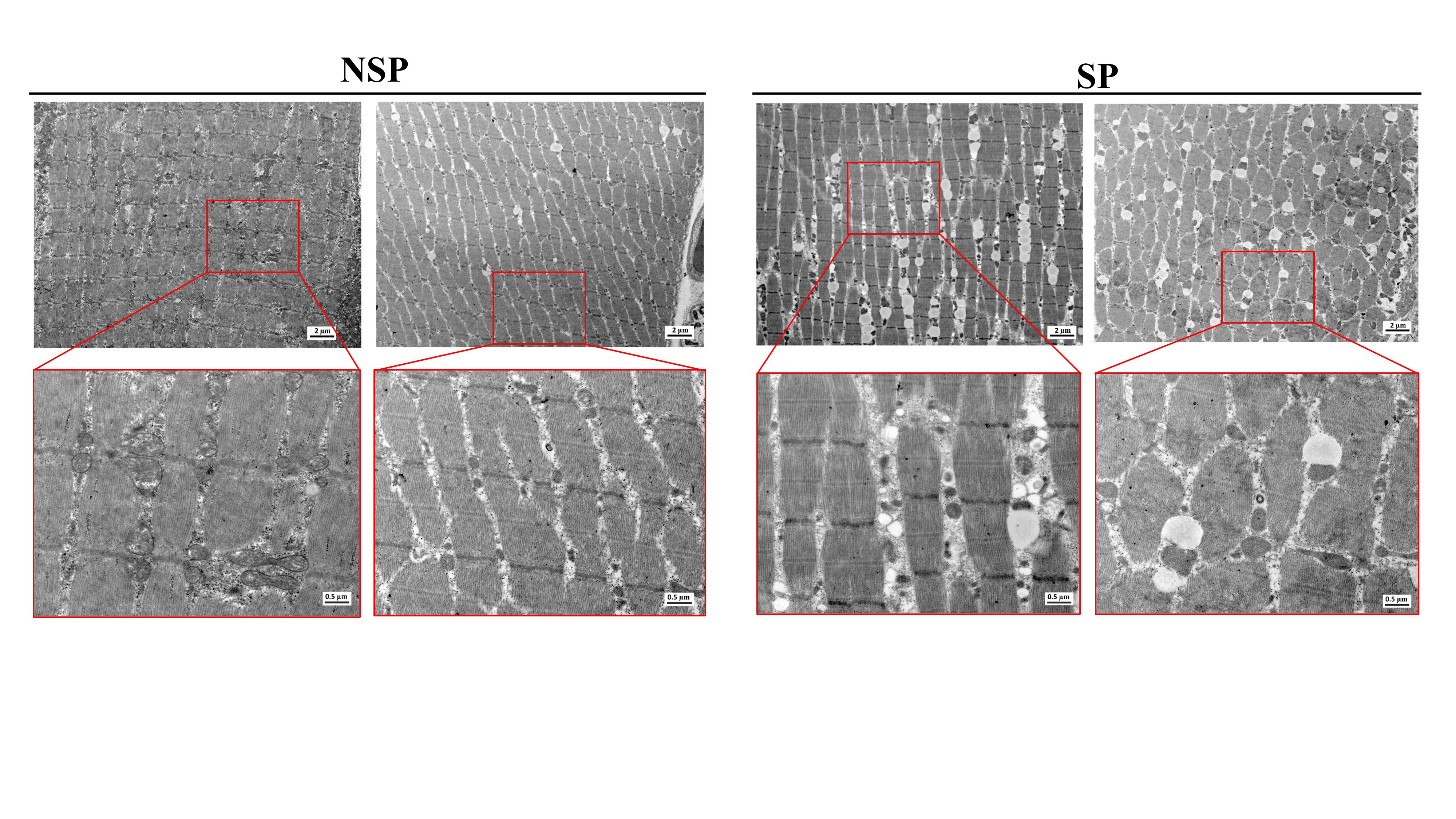

Supplement: Supplementary file 11 — Additional file 11. The representative images of TEM. [file 12877_2023_4152_MOESM11_ESM.jpg]
